# Telomeres and SIRT1 as Biomarkers of Gamete Oxidative Stress, Fertility, and Potential IVF Outcome

**DOI:** 10.3390/ijms25168652

**Published:** 2024-08-08

**Authors:** Anna Pańczyszyn, Ewa Boniewska-Bernacka, Iwona Wertel, Anna Sadakierska-Chudy, Anna Goc

**Affiliations:** 1 Institute of Medical Sciences, Department of Biology and Genetics, Faculty of Medicine, University of Opole, Oleska 48, 45-052 Opole, Poland; boniesia@uni.opole.pl (E.B.-B.); anna.goc@uni.opole.pl (A.G.); 2 Independent Laboratory of Cancer Diagnostics and Immunology, Medical University of Lublin, Chodźki 1, 20-093 Lublin, Poland; iwona.wertel@umlub.pl; 3 Department of Genetics, Faculty of Medicine and Health Sciences, Collegium Medicum, Andrzej Frycz Modrzewski Krakow University, Gustawa Herlinga-Grudzinskiego 1, 30-705 Krakow, Poland; asadakierska-chudy@afm.edu.pl

**Keywords:** sirtuin 1, telomeres, oxidative stress, fertility

## Abstract

The number of infertile couples undergoing in vitro fertilisation (IVF) has increased significantly. The efficacy of this procedure is contingent upon a multitude of factors, including gamete quality. One factor influencing gamete quality is oxidative stress, which leads to telomere damage and accelerates cellular ageing. Identifying new biomarkers that can predict the success of assisted reproduction techniques is a current relevant area of research. In this review, we discuss the potential role of SIRT1, a protein known to protect against oxidative stress and telomeres, which are responsible for genome stability, as biomarkers of gamete quality and assisted reproduction technique outcomes.

## 1. Introduction

A growing number of couples are undergoing assisted reproductive procedures. Many, despite numerous attempts, are unsuccessful. Several factors can affect gamete quality and in vitro fertilisation (IVF) outcomes. Researchers are currently investigating new biomarkers that can help predict the success of assisted reproduction techniques. Recently, scientific attention has been focused on sirtuin 1 (SIRT1), a protein that plays an important role in many cellular processes. Similarly, a comprehensive examination was conducted on telomeres, as they are crucial in determining the biological age of cells. A key factor influencing biological age and gamete quality is oxidative stress. Damage occurs within telomeric DNA under the influence of reactive oxygen species (ROS), potentially leading to premature cell ageing. SIRT1 is involved in protecting against oxidative stress and regulating cell senescence and DNA repair. This review aims to discuss the interaction between SIRT1 and telomeres and investigate whether SIRT1 and telomeres can serve as indicators of infertility and the efficacy of assisted reproduction techniques based on recent findings. The selection of SIRT1 and telomeres was deliberate, as recent research findings suggest a correlation between telomeres and SIRT1, which plays a significant role in gamete ageing and DNA damage response and potentially impacts fertility.

## 2. Sirtuin 1

Sirtuins belong to a conserved family of proteins, of which human cells have seven members (SIRT1—SIRT7). They are responsible for regulating many cellular processes, including the cell cycle, mitochondria functioning, ageing, metabolism, and oxidative stress [1]. Sirtuins are members of the class III histone deacetylases (HDACs), and their catalytic activity depends on NAD^+^ [2]. It recognises histone and non-histone proteins and catalyses the deacetylation of modified lysines.

SIRT1 is a key protein engaged in the ageing process located in the nucleus and cytoplasm and activated under oxidative stress. SIRT1 acts as a protein deacetylase by regulating many cellular pathways and is responsible for multiple functions. SIRT1 improves mitochondrial function and transcriptional activity via AMP-activated protein kinase activation [3]. It also regulates cellular metabolism by interacting with peroxisome proliferator-activated receptor gamma-coactivator-1α (PGC-1α), which plays a major role in gluconeogenesis and lipid metabolism [4,5]. Furthermore, SIRT1 aids in regulating the ageing process. On one hand, SIRT1 deacetylates Werner helicase (WRN) [6] and the NBS1 protein [7], which enables DNA damage repair. Wang et al. [8] demonstrated that in *Sirt1^−/−^* mice, chromosomal alterations frequently exist and DNA repair mechanisms are impaired. On the other hand, SIRT1 regulates apoptosis by interacting with several factors, such as transcription factors, E2F1, FOXO3a, and p53, and the nuclear factor-κB (NF-κB) complex [5].

## 3. Telomeres and Telomerase

**Figure 1:**
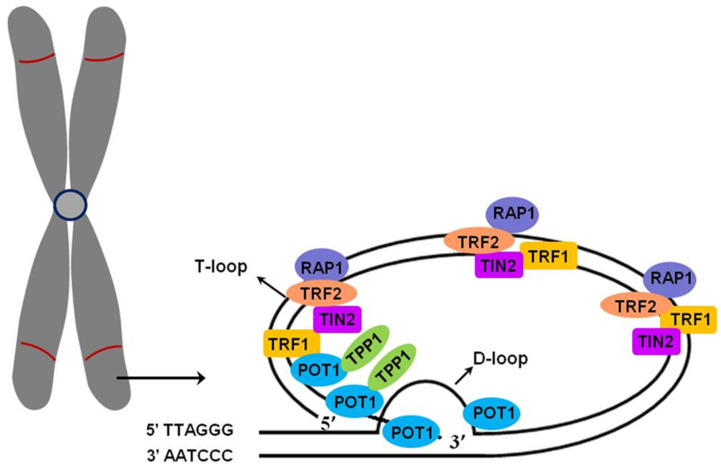
Telomere structure. At the end of each chromosome, there are multiple repeated (5′-TTAGGG-3′)_n_ sequences called telomeres. They comprise double-stranded T-loop and G-rich overhang, which is 50–400 bp and has a single strand at the 3′ end of the T-loop. The single strand invades the double-stranded DNA duplex of DNA, forming a D-loop. The telomere structure is stabilised and protected via the complex of proteins known as Shelterin complex.

Telomeres composed of repetitive (5′-TTAGGG-3′)_n_ sequences are located at the ends of chromosomes. Along with the protein complex Shelterin, telomeres protect chromosomes against genetic material loss, end fusion, and premature degradation. Telomere structures are unique, consisting of a double-stranded T-loop and G-rich overhang, which is 50–400 bp and has a single strand at the 3′ end of the T-loop. The single strand invades the double-stranded DNA duplex and forms a D-loop [9,10]. G-rich sequences can form G-quadruplexes (GQ), which are stable, four-stranded structures [11]. The protein complex Shelterin is composed of six subunits: TRF1, TRF2, Rap1, TIN2, TPP1, and POT1 (Figure 1) [12,13]. TRF1 and TRF2 directly bind to double-stranded telomeric sequences [14]. T-loop formation and the protection of the 3′ G-overhang depend on TRF2, and TRF1 is essential for efficient telomere replication [15]. The heterodimer POT1-TPP1 is associated with a single-stranded 3′ overhang [16] and protects telomeres via ATR kinase (ataxia telangiectasia and Rad3-related kinase) inhibition [17]. Additionally, TPP1, TIN2, and Rap1 bind to other Shelterin proteins. The Shelterin complex has several functions. Firstly, it caps telomeres and prevents them from binding to other proteins. Secondly, the ends of chromosomes are not detected as double-strand breaks when protected, which could activate the DNA damage response. Moreover, Shelterin proteins regulate telomere length (TL) [18]. POT1 inhibits telomere elongation by blocking telomerase access to the 3′ single strand, while TIN2, POT1, and TPP1 work together in telomerase activation to stimulate telomere elongation [19]. TIN2 participates in suppressing ATR and ATM (the ataxia-telangiectasia-mutated kinase) signalling by binding to heterodimer POT1-TPP1 [12]. It has been known that RNA polymerase II transcribes the C-rich sequences at the 3′ end of the chromosome to the long non-coding RNA called telomeric repeat-containing RNA (TERRA) [20]. TERRA is involved in a specific DNA-RNA hybrid structure termed R-loops at telomeres [21] and plays an important regulatory function by interacting with various proteins, such as the Shelterin complex. Furthermore, TERRA facilitates protein recruitment and enzymatic activities to the 3′ end of the chromosome participating in TL control [12].

Human TL typically ranges between 8 and 15 kb [22] and is highly variable [23]. In somatic cells, with every cell division, telomeres shorten by 50–200 bp due to the incomplete synthesis of the lagging strand during replication. Hence, telomere lengths limit the number of cell divisions and are called the “mitotic clock” [24]. Critically shortened telomeres lead the cell towards senescence since Shelterin proteins are unable to bind to DNA and perform their role in capping chromosomes and protecting genetic material [9,25]. The dynamics of telomere shortening can be influenced by external factors, such as oxidative stress, smoking, an unhealthy diet, and insufficient physical activity [24,26,27,28].

In specific cell types, the length of unprotected (uncapped) chromosomes is extended by the enzymatic complex, telomerase. A component of this complex is TERT, a catalytic unit acting as reverse transcriptase. The second important element is TERC, an RNA component that serves as a template to elongate the 3′ overhang of the telomeric G-rich strand. The enzyme is active in cells during foetal development. Telomerase remains inactive in somatic cells. However, its activity is maintained in the germ line, embryonic stem cells, and immune cells [29,30]. Telomerase reactivation in somatic cells often indicates carcinogenesis, as most cancer cells overexpress *hTERT* [31].

## 4. The Role of SIRT1 in Sperm Cell and Oocyte Development

The role of SIRT1 in spermatogenesis and oogenesis has been extensively investigated. SIRT1 is crucial for normal spermatogenesis. Research based on the *Sirt1*^−/−^ mouse model indicated that SIRT1-deficient male mice have relatively smaller testes and suffer from infertility due to poor spermatogenesis and abnormal sperm maturation [32,33]. Immunohistochemical techniques demonstrated that SIRT1 is present in the nuclei of spermatogenic cells at different stages of spermatogenesis [34], and the absence of SIRT1 results in morphological abnormality in sperm cells and more frequent spermatocyte and spermatid apoptosis [32]. Male germ cell apoptosis in *Sirt1*^−/−^ mice was associated with p53 acetylation and lower-level genome integrity. SIRT1 affects spermatogenesis by regulating the transcription of several genes, including sumoylation-related genes, which are known as regulators of germ cell proliferation, heterochromatin remodelling, and nuclear morphology changes during spermatogenesis [32,35,36]. In addition, SIRT1 deacetylates PGC-1α, activating SIRT3 transcription, which, in turn, enhances critical processes for normal spermatogenesis, such as oxidative stress defence, glucolytic activity, and lactate synthesis [33]. The significance of SIRT1 in acrosome biogenesis [37,38] and histone to protamine replacement has also been documented [34]. The primary mechanism by which SIRT1 regulates spermatogenesis is through modulating the hypothalamic–pituitary–gonadal (HPG) axis [39]. A reduced production of the gonadotropin-releasing and follicle-stimulating hormones in *Sirt1*^−/−^ mice was observed. In addition, the inhibition of luteinising hormone secretion was noticed; therefore, Leydig and Sertoli cell maturation was impaired [40].

**Figure 2:**
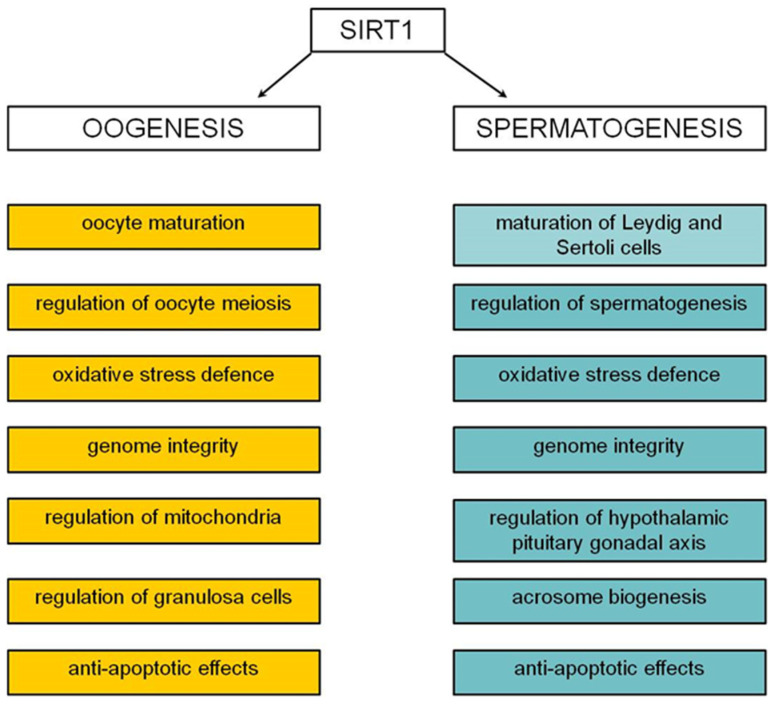
The role of SIRT1 during oogenesis and spermatogenesis.

Oogenesis is also regulated via SIRT1 in numerous ways. Iljas et al. [41] used the *Sirt1*^−/−^ mouse model to demonstrate that the ovarian reserve and ability to produce mature oocytes were not affected by a SIRT1 deficiency but caused premature sterility in *Sirt1*^−/−^ females compared to wild-type females. The authors propose that defects appear in elderly *Sirt1*^−/−^ females due to age-related changes, such as reduced NAD^+^ and lower levels of sirtuins, which compromise mechanisms that can compensate for SIRT1 loss in younger oocytes. SIRT1 plays a role in meiosis progression. For instance, SIRT1 regulates a nuclear E2-related factor 2 (NRF2) that regulates cyclin B1 expression and is essential to oocyte maturation and spindle organisation [42]. During oogenesis, SIRT1 changes its localisation. Ferreira et al. [43] revealed the nuclear presence of SIRT1 and SIRT2 in the germinal vesicle stage and the colocalisation of SIRT1, 2, and 3 with both metaphase-I and metaphase-II spindles. Nevoral et al. [44] observed that spindle-specific SIRT1 action decreases α-tubulin acetylation and suggested that SIRT1 plays dual spatiotemporal action in oocytes, which can be readily switched from the epigenetic to the non-epigenetic mode of action according to the meiosis progress. Apart from regulating meiosis, SIRT1 protects oocytes against oxidative stress by promoting the activities of transcription factor FOXO3 and superoxide dismutase (SOD) [45]. In postovulatory oocytes, SIRT1 delays ageing by reducing the changes in histones’ H3K9ac, H3K4 methylation, ROS accumulation, spindle morphology, and mitochondrial functions [46]. The role of SIRT1 in regulating cells surrounding oocyte has been extensively studied. It was established that SIRT1 regulates granulosa cell differentiation and luteinisation [47,48] and affects mitochondria function. After SIRT1 silencing, *Mitofusin-2* and *Atg5* expressions, genes associated with mitochondria function, significantly decreased [49]. Han et al. [50] demonstrated that SIRT1 protects granulosa cells against apoptosis by activating the ERK1/2 (extracellular signal-regulated protein kinase) and inhibiting nuclear factor NF-κB signalling pathways. Moreover, SIRT1 can directly regulate Bax and Bcl-xL expressions, proteins that regulate granulosa cell apoptosis [51,52] and apoptosis through the SIRT1/p53 axis [53]. Figure 2 summarises the most important SIRT1 functions in oogenesis and spermatogenesis.

SIRT1 plays a role in regulating gamete development, acting to protect against oxidative stress and apoptosis and enhance genome stability. During oogenesis, it regulates oocyte meiosis and granulosa cell luteinisation. During spermatogenesis, it is involved in acrosome maturation, Sertoli and Leydig cells, and hormone regulation.

## 5. Telomeres in Sperm Cells

Spermatogenesis is a continuous process occurring in the testicles from puberty influenced by male hormones. A low level of testosterone has a positive impact on the leukocyte telomere length (LTL), while dihydrotestosterone is positively associated with LTL [54]. The length of sperm cell telomeres is important for offspring telomeres. Over the last few decades, it has been observed that parents with longer telomeres have progeny with longer telomeres, which diminish the risk of age-related diseases [55,56]. Previous reports indicated that the sperm telomere length (STL) ranges from 10 to 20 kb and mature spermatozoa contain longer STL compared to somatic cells [57,58]. The telomerase is mostly active during the early stages of spermatogenesis in determining STL, whereas its activity decreases in spermiogenesis [59]. In contrast to somatic cells, STL becomes longer as men age. Eisenberg et al. [60,61] indicated that older paternal age at conception predicts longer offspring TL.

However, high STL heterogeneity can be affected by various factors, such as oxidative stress, diet, and smoking [62]. Infertile men have shorter telomeres than fertile men [63], which correlates with DNA fragmentation and lipid peroxidation. Abnormal protamination has also been associated with short STL [64]. Oxidative stress is another important factor correlated with STL widely discussed in the literature. Reactive oxygen species negatively affects STL [65,66] and is related to abnormal sperm parameters such as DNA fragmentation and chromatin condensation defects [67]. Although acute oxidative stress is detrimental to telomeres [68], mild oxidative stress is beneficial for STL and leads to telomere lengthening [69], but the exact mechanism of this phenomenon remains unclear. Recently, Moazamian et al. [65] presented a hypothetical mechanism of telomere lengthening with age in men that is associated with oxidative stress. Oxidative stress destabilises the G-quadruplex (GQ) structure that normally inhibits telomerase, leading to telomere elongation. In other cells, telomerase activity is restricted to the period in which AP endonuclease-1 (APE1) has not yet completed the repairing and reorganising of the GQ structure. As sperm cells lack APE1, it is possible that telomerase activity could remain unchecked since the GQ structure cannot be restored.

## 6. Telomeres in Oocytes

Oogenesis is the process that occurs during female embryogenesis. Primordial germ cells undergo mitotic divisions, resulting in oogonia production. Oogonia undergo mitosis to generate primary oocytes, which subsequently enter meiosis and undergo arrest at prophase I. This stage of arrest can persist until the onset of puberty or even to menopause [70]. Oocyte telomere lengths (OTLs) are determined during early oogenesis when telomerase has the highest activity. Its activity decreases during maturation, i.e., at late stages of oogenesis [71,72]. Due to inactive telomerase, oocyte telomeres are shorter than somatic telomeres. The lack of germline stem cells in women’s ovaries results in the accelerated ageing of oocytes, which, in turn, leads to genomic instability, abnormal spindle formation, and abnormal embryo production [73]. The oocyte telomere lengths can be affected by several factors, including oxidative stress, smoking, and obesity. However, the most significant factor is advanced maternal age [74,75,76]. Advanced reproductive age is associated with oocyte telomere shortening due to ROS accumulation, which causes DNA damage during oocyte meiotic arrest [77]. Shorter telomeres have been observed in different diseases of the reproductive system such as ovarian cancer [78], endometriosis [79], premature ovarian failure (POF) [80], and polycystic ovarian syndrome (PCOS) [81]. Furthermore, Pollack et al. [82] indicated that women who have at least one live birth have longer leukocyte telomeres than women who experienced biochemical pregnancy or miscarriage.

## 7. SIRT1 and Telomeres in Gamete Protection against Premature Ageing Caused by Oxidative Stress

Oocyte quality declines with age. A possible factor that accelerates oocyte and ovary ageing is oxidative stress, which arises from an imbalance between ROS production and the antioxidative cell defence mechanisms, mainly due to mitochondrial dysfunction [83]. Enzymes such as xanthine oxidase (XO), NADPH oxidases (NOXs), and mitochondrial electron transport chain (ETC) can generate ROS, including highly reactive free oxygen radicals, oxygen ions, and peroxides [84]. Excessive ROS production results in extensive DNA damage, including base modifications, DNA cross-links, single-strand or double-strand breaks (DSBs), which, in turn, promotes cell death [85]. Telomeres are vulnerable to ROS that causes single-strand breaks [86], but the most frequent telomeric DNA modification is 8-oxo-7,8-dihydroguanine (8-oxoG) because of the high level of guanine in their sequence [15]. The presence of 8-oxoG in telomeric DNA also changes the ability of TRF1 and TRF2 to bind DNA [87]. The association between oxidative stress and telomere shortening is well documented in the literature [25,88,89]. Coluzzi et al. [90] suggested that oxidative damage to telomeric DNA leads to telomere shortening and increases chromosome instability via telomere end fusion. Furthermore, the telomere damage mechanism include replication fork arrest, epigenetic changes in histone, and the reduction in TRF1 and TRF2 [15]. Hence, telomeric 8-oxoG accumulation drives the telomeric crisis, which leads to cell apoptosis if not repaired [68]. Oxidative stress declines oocyte quality and sperm cells and fastens gamete apoptosis. SIRT1 protects oocytes from oxidative stress in several ways [91]. Firstly, SIRT1 regulates the transcription factor, FOXO, which is responsible for upregulating antioxidant enzymes, such as catalase (CAT) and mitochondrial SOD [92,93]. Secondly, SIRT1 deacetylates NRF2, which is a main regulator of enzymatic and non-enzymatic antioxidant defences [94]. Furthermore, SIRT1 participates in maintaining telomere homeostasis. ROS accumulation causes telomeres damage, resulting in the activation of SIRT1-PGC-1α axis signalling. DNA repair demands NAD^+^, which, in turn, leads to a loss of SIRT1 activity [76,95]. Telomere dysfunction can also cause PGC-1α/β repression and lead to a decrease in the number of mitochondria, which is mediated by p53 [96]. In turn, p53 deacetylation via SIRT1 suppresses its apoptotic effects, while p53 hyperacetylation supposedly mediates the anticancer effects of sirtuin inhibitors [40,97]. Mild oxidative stress conditions can increase SIRT1 expression, thereby triggering the response to changes in the cell redox status. Conversely, severe oxidative stress increases SIRT1 degradation, ultimately leading to apoptosis [40]. Iljas et al. [41] demonstrated that SIRT1 deletion in mouse oocytes results in oocyte quality decline and increased oxidative stress in embryos. SIRT1 can delay oocyte ageing by improving mitochondrial function [98] and promote the biogenesis of mitochondria via PGC-1α activation in the presence of NAD^+^ [99].

ROS accumulation, similar to that observed in oocytes, can have a detrimental effect on the production and quality of sperm cells [100]. ROS leads to morphological alterations in the seminiferous epithelium, cytoplasmic vacuolisation, and apoptosis in both germinal and Sertoli cells [101,102]. During spermatogenesis, SIRT1 triggers antioxidant activities mainly by activating PGC-1α. According to Liu et al. [37], decreased SIRT1 expression leads to defects in acrosome formation, resulting in abnormal sperm morphology. Sperm telomere shortening due to ROS exposure affects spermatogenesis through segregation errors, cell cycle delays, reduced regenerative capacity, and cell death [64]. SIRT1 protects telomeres against ROS by inducing the repair mechanism and *hTERT* expression. In SIRT1 knock-out mice, single- and double-strand DNA breaks were frequently observed [39]. A recent study conducted by Dhillon et al. [103] demonstrated an inverse correlation between SIRT1 and SIRT3 levels and oxidative stress biomarkers in seminal plasma. In this study, infertile men had significantly shorter TLs in sperm cells and leukocytes and a higher sperm DNA fragmentation index, which could be caused by ROS.

Studies indicated that SIRT1 participates in DNA damage responses caused by ROS via histone and non-histone protein post-transcriptional modifications [85]. Modified telomeric bases are repaired via a base excision repair system (BER). During incomplete BER, ssDNA intermediates can persist or accumulate; as a result, they can lead to replication fork collapse and subsequent DSB formation [104,105]. A recent study by Barnes et al. [106] indicated that oxidative stress facilitates rapid senescence by generating oxidative base lesions that result in replication-dependent telomere fragility and dysfunction. According to Barnes et al., acute 8-oxoG production fails to shorten telomeres and, instead, generates fragile sites and DNA synthesis in telomeres. Furthermore, 8-oxoG activates ATM and ATR kinases and the downstream effectors p53 and p21. Fouquerel et al. [68] demonstrated that persistent 8-oxoG stimulation leads to reduced TL and inhibits cell proliferation. Other studies indicated that mild oxidative stress has the opposite effect, resulting in telomere lengthening [69]. It has been widely acknowledged that 8-oxoG can destabilise GQ structures and has the potential to activate telomerase and stimulate telomere extension [68]. Recent findings suggest that, in cancer cells, 8-oxoG stimulates the alternative lengthening of telomere (ALT) in the G2 phase of the cell cycle [107].

**Figure 3:**
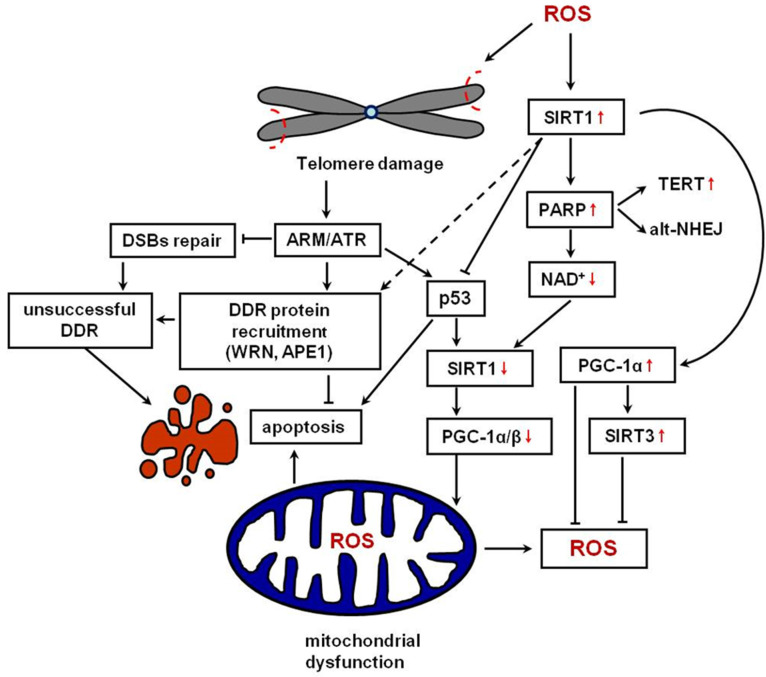
The interplay between telomeres and SIRT1 under oxidative stress. ROS leads to telomere damage and shortening, which activates ATM or ATR kinase. Kinases recruit DNA-damage-response proteins. In the case of unsuccessful repair, the cell undergoes apoptosis. The SIRT1 level rises under oxidative stress. SIRT1 also participates in the DNA damage response reaction by inducing PARP, which stimulates TERT and DNA repair via the alt-NHEJ mechanism. SIRT1 also delays apoptosis by inhibiting p53. The arrows indicate increasing or decreasing a given factor.

Proteins specifically involved in 8-oxoG repair constitute the GO system. An 8-oxoG:C base pair is recognised and excised by the OGG1 glycosylase that subsequently generates an apurinic (AP) site. Then, APE1 cleaves the AP site, and the correct G:C base pair is restored [105,108]. SIRT1 deacetylates APE1, allowing APE1-XRCC1 complex formation and the stimulation of APE1 activity [109]. Thus, as indicated by Elamin et al. [110], SIRT1 decreases 8-oxoG. Furthermore, SIRT1 and transcription factor E2F1 are involved in a signalling cascade initiated by a molecular sensor of DNA single breaks, known as PARP1 (poly(ADP-ribose) polymerase 1). Both proteins regulate the *Arf* gene, a tumour suppressor involved in p53 signalling [111]. Telomere dysfunction also leads to p53 activation. In contrast, p53 represses sirtuins through transcriptional and post-transcriptional mechanisms. SIRT1 is repressed through miRNA-34a. In turn, mitochondrial sirtuins, such as SIRT3, SIRT4, and SIRT5, are regulated via PGC-1α/β. Augmented NAD^+^ levels through NAD^+^ precursors results in an increase in sirtuin activity, stabilises telomeres, and mitigates p53 in a partially SIRT1-dependent manner. Because SIRT1 is known to activate PGC-1α through deacetylation, SIRT1 repression directly contributes to PGC-1α impairment. Low levels of SIRT1 and SIRT6 can further destabilise telomeres and lead to ageing. The interplay between telomeres, p53, and sirtuins accelerates the decline of damaged cells, especially if telomere dysfunction alone does not result in apoptosis (Figure 3) [112,113]. 8-oxoG repair can be challenging for sperm cells. Although sperm cells contain OGG1, they do not have APE1, which maintains abasic sites during DNA repair [114].

DSBs are the most severe DNA lesions. Typically, they can be repaired via two mechanisms: homologous recombination (HR) and non-homologous end joining (NHEJ). In the S-phase of the cell cycle, DSBs in telomeres can be repaired with alternative non-homologous end joining (alt-NHEJ) [115]. However, if DSBs are present in telomeres during mitosis, Shelterin proteins prevent the activation of three DNA damage response enzymes (ATM, ATR, and PARP1), blocking DSB repair pathways and inducing senescence [116,117,118].

## 8. The Length of Telomeres as a Prognostic Factor of Fertility and IVF Outcomes

As mentioned above, shorter TLs in sperm cells have the potential to diminish the likelihood of achieving reproductive success. Therefore, researchers are looking for new critical markers in estimating the chances of obtaining offspring from individuals with reduced fertility undergoing assisted reproductive techniques. Telomeres as biomarkers have been extensively explored in various diseases, such as cardiovascular diseases [119] and cancers [120,121]. A large body of data suggests that they may also serve as a marker of infertility and a prognostic tool for the effectiveness of assisted reproductive methods [122]. Table 1 presents the main studies that have analysed the association between the TL of leukocytes, cumulus, and granulosa cells with female fertility and chances of pregnancy. The published results do not provide a clear answer whether TL can serve as a prognostic marker for IVF outcomes. Some studies have found a positive correlation between longer TL, good oocyte quality, and chances of pregnancy [123,124,125], but others have not confirmed this [126,127,128]. The challenges inherent to analysing OTL are twofold: firstly, the limited accessibility of the oocytes and, secondly, the ethical considerations for using mature oocytes. Hence, the discrepancies between specific studies are due to the differences in cells being taken for analysis. The granulosa cells that surround oocytes can be readily accessed during IVF procedures. The results indicate that granulosa cells’ telomeres and telomerase expression may reflect the telomere length of the oocyte [123]. The negative correlation between the telomere length in granulosa cells and the aneuploidy rate in the young age group suggests that TL may be a useful indicator for selecting competent oocytes [129]. Advanced female age is a key factor in reproductive failure. It has been shown that women of advanced age who undergo IVF procedures have shorter telomeres and their pregnancies end with miscarriage more frequently [130] or the embryos show aneuploidy [131].

The role of TL in predicting fertility has been better documented in sperm than oocytes due to the easier acquisition of research material. Table 2 summarises the most important results of studies examining the length of telomeres in sperm or leukocytes and their relationship with fertility and pregnancy rate. Short telomeres may reflect spermatogenesis and sperm cell quality, which is potentially associated with fertility. Several studies reported that STL is correlated with sperm parameters such as sperm count [143,144,145], motility [58,63,146], and vitality [63], while others did not confirm these observations [62,134,147,148]. The discrepancies between specific reports may be due to the different methods used for TL measurement. For instance, fluorescence in situ hybridization (FISH) allows for measuring the length of telomeres in individual telomeres of a single cell, while quantitative real-time PCR (qPCR) is dedicated to measuring the relative TL on the genome. Q-PCR is commonly used for large-scale epidemiological studies due to its high-throughput nature but is less precise than other methods [149]. Other factors that influence TL measurement results are age and the above-mentioned lifestyle. The type of cell used for TL analysis is also important. Easily accessible leukocytes may not be good indicators of the telomere length of other tissues. Despite the strong correlation between the telomere lengths of leukocytes and numerous other tissues, reproductive organs tend to possess longer telomeres than leukocytes [150].

So far, many studies separately analysed telomere lengths in infertile men or women who underwent assisted reproduction techniques. Thilagavathi et al. [130] analysed the TLs of couples experiencing idiopathic recurrent pregnancy loss and found that TL was significantly lower in both men and women compared to controls. Thus, shorter TLs for both parents can reduce the likelihood of conceiving. Telomere lengths seem to be an important factor associated with the likelihood of implantation. Recently, Chien et al. [151] analysed the telomeres from 965 blastocysts of 164 patients, respectively, and found that TL is strongly associated with successful pregnancy outcomes. The study also confirmed that advanced age of women is correlated with a higher percentage of aneuploid embryos. Furthermore, a recent meta-analysis by Yuan et al. [152] confirmed a positive correlation between STLs and clinical pregnancy outcomes.

**Table 2:** Review of studies on telomere length, male fertility, and IVF outcomes.

Samples	N	Age	Method	IVF	Main Findings	Ref.
Sperm cells Leukocytes	81	18–19	qPCR	−	STL and LTL are positively correlated, but sperm telomeres are longer than leukocyte telomeres A significant positive correlation between STL and total sperm number was found STL was significantly lower in oligozoospermic than in normozoospermic men	[144]
Leukocytes	45	N.A.	qPCR	−	Shorter telomere length in both men and women may be associated with early pregnancy loss Negative association between seminal volume and TL Positive association between TL and fragmentation index of sperm	[130]
Sperm cells	45	21–49	Q-FISH	+	No association between STL, male fertility, and IVF outcome Sperm telomere length increased with male age	[134]
Sperm cells	10	32–47	-qPCR	+	Longer STS in older men than in younger STS positively correlated with age and semen parameters	[153]
Sperm cells	105	31.2 ± 6.1	qPCR	−	Telomere length was positively correlated with semen sperm count	[143]
Sperm cells	418	N.A.	qPCR	+	STL was positively associated with embryo quality in IVF STL was positively correlated with the age of patient and total sperm count	[154]
Sperm cells	73	31–52	qPCR	+	Sperm telomere length positively correlated with sperm count Shorter STL in oligospermic men in comparison to normozoospermia group Negative association between STL and IVF outcome	[145]
Sperm cells	214	31.71 ± 4.45 32.22 ± 4.0	qPCR	−	Infertile men had shorter telomeres comparing to control	[69]
Sperm cells	100	34.0 + 8.6	qPCR	−	Positive correlation between STL and motility, vitality, and protamination STL negatively associated with sperm DNA fragmentation	[63]
Sperm cells	26	29–42	FISH	−	Telomeres were shorter among the participants with sub-fertile sperm compared to fertile sperm	[155]
Sperm cells	30	N.A.	Q-FISH	−	TL was shorter in spermatozoa from couples who never achieved a pregnancy compared to couples who did achieve at least one natural pregnancy	[58]
Sperm cells	60	18–35	qPCR	+	There was no significant correlation between STL and fertilisation rate STL measurement is not useful to predict reproductive outcomes in ICSI cycles using donor semen	[156]
Sperm cells	20	38.10 ± 4.17 40.11 ± 3.14	qPCR	+	Reduced telomere length in spermatozoa of infertile men with previous failed/low fertilisation compared with fertile individuals	[157]
Sperm cells	65	35.53 ± 4.48	qPCR	+	STL is positively correlated with in vitro fer- tilisation success No associations were observed between STL and sperm count, concentration, or pro- gressive motility TL did not associate with age, BMI, health, or lifestyle factors	[158]
Leukocytes	31 (I) 100 (F)	23–54	Q-FISH	−	Shorter TL in infertile group Infertile group had more chromosome loss and telomere doublet formation than controls	[140]
Sperm cells	78	29–48	qPCR	+	No significant correlations were found between selected STL and sperm quality No relation was observed between selected STL and clinical outcomes	[148]
Sperm cells Leukocytes	57	20–50	qPCR	−	LTL and STL were significantly shorter in infertile men compared with fertile individuals Significant associations between telomere length with sperm concentration, DNA fragmentation, and lipid peroxidation	[64]
Sperm cells Leukocytes	65	39.6 ± 5.4 36.1 ± 6.8	qPCR	+	STL was reduced in patients that underwent ART (assisted reproductive technologies) STL was positively correlated with embryo quality	[159]
Sperm cells Leukocytes	239	18–59	qPCR	−	There was no association between STL and sperm parameters No associations between sperm parameters and STL nor LTL	[147]
Sperm cells Leukocytes	77	<25 >40	Q-FISH	−	Younger OAZ (oligoasthenozoospermia) individuals showed significantly shorter mean telomere length	[146]
Sperm cells	34	18–60	qPCR	+	Sperm motility was negatively correlated with TL No correlation was identified between pregnancy outcomes and TL	[62]
Spermatogenic cells	30	N.A.	Q-FISH	+	Positive correlation between TL of spermatogonia and spermatocytes and IVF outcome	[160]
Sperm cells	60	<40	qPCR	−	TL of teratozoospermia samples is nearly 3 times shorter than in normal samples	[161]
Leukocytes Sperm cells	272 (O) 251 (F)	32.72 ±4.91 31.52 ± 4.35	qPCR	−	Shorter STL and LTL in oligozoospermic men Lower levels of oxidative stress biomarkers and DNA fragmentation in oligozoospermic men	[103]

Abbreviations used in the table: I—infertile group; F—fertile group; O—oligozoospermic group; N.A.—not available.

## 9. Can SIRT1 Be a Potential Indicator of IVF Success?

Previous studies suggested that SIRT1 levels in follicular fluid and serum have the potential to serve as an indicator of good oocyte quality and a prognostic factor for IVF outcome. Ovary overstimulation led to significantly higher levels of SIRT1 in pregnant than non-pregnant women. Furthermore, serum SIRT1 was related to pregnancy [162,163]. Recently, Jingyun et al. [164] found that serum and follicular fluid levels of SIRT1 decreased with age in women undergoing IVF or intracytoplasmic sperm injection (ICSI). Patients over the age of 40 had significantly lower levels of SIRT1, which was associated with lower implantation and clinical pregnancy rates compared to younger women. Furthermore, oxidative stress markers such as SOD and plasma glutathione peroxidase (GSH-Px) were positively associated with SIRT1 in these patients. Similar results were obtained by Nasiri et al. [165] who analysed SIRT1 and SIRT3 levels in the seminal plasma of normozoospermic and asthenoteratozoospermic men. The asthenoteratozoospermic group had significantly lower levels of these proteins, which were also negatively correlated with oxidative stress and DNA fragmentation.

Based on the available research, increasing SIRT1 levels may potentially improve IVF success rates, particularly for older women or those with oxidative stress issues. It must be acknowledged that the research conclusions presented above are, as of yet, only preliminary and based on trials involving relatively small research groups. The observations of the aforementioned authors indicate an important role for SIRT1 in the proper course of gametogenesis and fertilisation. However, further in-depth research is required to demonstrate the usefulness of measuring SIRT1 concentration in the process of predicting the effectiveness of IVF. It would be beneficial to simultaneously analyse the SIRT1 level and telomere length of sperm and granulosa cells in the sera of couples undergoing an IVF procedure. This approach could provide further insights into the potential role of SIRT1 and telomere length as markers of IVF efficacy.

## 10. Conclusions

In this review, we considered the importance of SIRT1 and telomeres in fertility to determine whether SIRT1 and telomeres could serve as prognostic indicators of the efficacy of assisted reproductive techniques. To our knowledge, this is the first review that gathered information about telomeres and SIRT1, their interaction, and their possible roles as biomarkers of fertility and IVF efficacy. Many cellular processes are affected by SIRT1, including oxidative stress response regulation, cell ageing, and DNA repair. Oxidative stress leads to DNA damage, telomere shortening, and cellular senescence; therefore, it is a critical factor in premature gamete ageing. By activating DNA damage repair and telomerase, SIRT1 protects telomeres from shortening and delays cell apoptosis. Telomere lengths seem to be indicators of the chance of conception since infertile individuals have shorter telomeres. SIRT1 works with telomeres, stimulating DNA repair and delaying apoptosis. Hence, it is a critical fertility regulator in both males and females by maintaining germ cell function, protecting against oxidative stress, and modulating key reproductive pathways. SIRT1 levels can indicate gamete quality and the likelihood of fertilisation.

The results of the studies discussed in the above review indicate that SIRT1 and telomeres play a critical role in gametogenesis and impact male and female fertility. Nevertheless, further investigation is required to ascertain the clinical utility of these measurements, with a view to standardise methodologies and evaluate their predictive capacity.

## Figures and Tables

**Table 1:** Review of studies on telomere length, female fertility, and IVF outcomes.

Samples	N	Age	Method	IVF	Main Findings	Ref.
Granulosa cells	12 (OI) 42 (H)	30–37 23–37	qPCR	+	Shorter TL in women with ovarian insufficiency than in controls	[132]
Leukocytes	34 (POF) 95 (AM) 108 (H) 46 (F)	21–50 24–45 17–55 37–54	qPCR	−	Women with POF had longer LTL	[133]
Oocytes	32	25–42	qPCR	+	Longer telomeres in immature oocytes than in mature oocytes No association between oocyte TL with clinical parameters and IVF outcomes	[134]
Oocytes Cumulus cells	350 (OCC)	29 ± 3.4 41.4 ± 1.9	qPCR	+	TL of cumulus cells from mature oocyte were longer than TL of cumulus cells from immature oocyte TL of cumulus cells may be a marker for selecting highly competent oocytes and good-quality embryos	[123]
Leukocytes	20 (FC) 25 (IC)	<40 (f) 18–40 (FC)	qPCR	−	Shorter TL in both men and women may be associated with early pregnancy loss	[130]
Granulosa cells	76	23–38	qPCR	+	TL did not differ significantly between the pregnant and non-pregnant groups Telomerase activity is a better predictor of pregnancy outcomes following IVF treatment than TL	[126]
Leukocytes	698 (PCOS) 611 (H)	26.42 ± 0.19 28.77 ± 0.27	qPCR	−	Women with PCOS had shorter LTLs than the controls	[135]
Leukocytes	20 (I) 10 (H)	29.3 (4.3) 28.6 (3.4)	qPCR	+	Women undergoing IVF had statistically significant higher levels of CFD (Cell –free DNA) and shorter TL compared to healthy controls.	[125]
Leukocytes	150 (PCOS) 127 (H)	27.22 (5.99) 29.36 (5.18)	qPCR	−	LTL did not differ between groups Age and BMI had no significant effect on LTL The inflammatory biomarkers CRP and homocysteine were negatively correlated with LTL in patients with PCOS	[136]
Leukocytes	40 (PCOS) 35 (H)	24.78 ± 3.54 26.33 ± 2.15	qPCR	−	LTLs were significantly longer in the PCOS group than in the control group	[137]
Leukocytes Granulosa cells	75 (PCOS) 81 (IVF)	28.36 ± 2.55 28.09 ± 2.26	qPCR	+	No significant difference was found in the LTL between controls and PCOS patients PCOS women had longer LTL than controls	[138]
Cumulus cells (CC) Leukocytes (L) Granulosa cells (GC)	35	18–33	qPCR	−	No correlation was observed between TL measurements of L vs. CC and L vs. GC CCTL was significantly higher than LTL (1.54-fold) CC from mature follicles have significantly longer telomeres than L	[139]
Leukocytes	19 (I) 100 (F)	23–54	Q-FISH	−	Shorter TL in infertile group Infertile group had more chromosome loss and telomere doublet formation than controls	[140]
Leukocytes (L) Cumulus cells (CC)	175	35 ± 4	qPCR	+	CC relative telomere length is significantly longer than the relative LTL Leukocyte TL was associated with embryo aneuploid	[131]
Leukocytes	30 (F) 30 (I)	44.23 ± 1.40 44.9 ± 1.35	TRF	−	No apparent effects of pregnancy, delivery, or parity on TL	[127]
Leukocytes Granulosa cells	60 (Y) 50 (O)	33.5 ±3.1 40.7 ± 1.9	qPCR	−	mtDNA copy number and TL were positively correlated in both leukocytes and granulosa cells TL was significantly shorter in granulosa cells than leukocytes in both groups TL in the granulosa cells was negatively correlated with the aneuploidy rate in the young age group	[129]
Leukocytes	181	37–39	qPCR	+	No association between TL and IVF outcome	[128]
Granulosa cells	160	23–45	qPCR	+	Shorter TL and increased TERT expression in older women Inverse correlation between TERT and oocyte yield The pregnancy of women >35 years old was 20% lower than those of women <35	[124]
Granulosa cells (GC) Follicular fluid (FF)	102	35.11 ± 4.89	qPCR	+	TLs were independent of telomerase activity both in GCs and FF	[141]
Granulosa cells	51	20–34 40–45	qPCR	+	Granulosa cells’ telomere length of the young normal responder was found to be significantly longer than young poor ovarian responder or elderly patients	[142]

Abbreviations used in the table: AM—group after miscarriage; f—female; F—fertile group; FC—fertile couples; H—healthy donors; I—infertile group; IC—infertile couples; IVF—group undergoing IVF; IVF—in vitro fertilisation; O—older group; OCC—oocytes–cumulus complex; OI—group with ovarian insufficiency; PCOS—group with polycystic ovary syndrome; POF—group with premature ovarian failure; Y—younger group; Q-FISH—quantitative fluorescence in situ hybridisation; qPCR—quantitative real-time PCR, TRF—telomere restriction fragment analysis.
